# The good and the bad of T cell cross-reactivity: challenges and opportunities for novel therapeutics in autoimmunity and cancer

**DOI:** 10.3389/fimmu.2023.1212546

**Published:** 2023-06-19

**Authors:** Cécile Gouttefangeas, Reinhild Klein, Ana Maia

**Affiliations:** ^1^ Department of Immunology, Institute for Cell Biology, University of Tübingen, Tübingen, Germany; ^2^ Cluster of Excellence iFIT (EXC2180) “Image-Guided and Functionally Instructed Tumor Therapies”, University of Tübingen, Tübingen, Germany; ^3^ German Cancer Consortium (DKTK) and German Cancer Research Center (DKFZ) partner site Tübingen, Tübingen, Germany; ^4^ Department of Hematology, Oncology, Clinical Immunology and Rheumatology, University Hospital Tübingen, Tübingen, Germany

**Keywords:** T cell receptor, cross-reactivity, autoimmunity, cancer, immunotherapy

## Abstract

T cells are main actors of the immune system with an essential role in protection against pathogens and cancer. The molecular key event involved in this absolutely central task is the interaction of membrane-bound specific T cell receptors with peptide-MHC complexes which initiates T cell priming, activation and recall, and thus controls a range of downstream functions. While textbooks teach us that the repertoire of mature T cells is highly diverse, it is clear that this diversity cannot possibly cover all potential foreign peptides that might be encountered during life. TCR cross-reactivity, i.e. the ability of a single TCR to recognise different peptides, offers the best solution to this biological challenge. Reports have shown that indeed, TCR cross-reactivity is surprisingly high. Hence, the T cell dilemma is the following: be as specific as possible to target foreign danger and spare self, while being able to react to a large spectrum of body-threatening situations. This has major consequences for both autoimmune diseases and cancer, and significant implications for the development of T cell-based therapies. In this review, we will present essential experimental evidence of T cell cross-reactivity, implications for two opposite immune conditions, i.e. autoimmunity vs cancer, and how this can be differently exploited for immunotherapy approaches. Finally, we will discuss the tools available for predicting cross-reactivity and how improvements in this field might boost translational approaches.

## 1 Introduction: basics on TCR cross-reactivity

**Figure 1:**
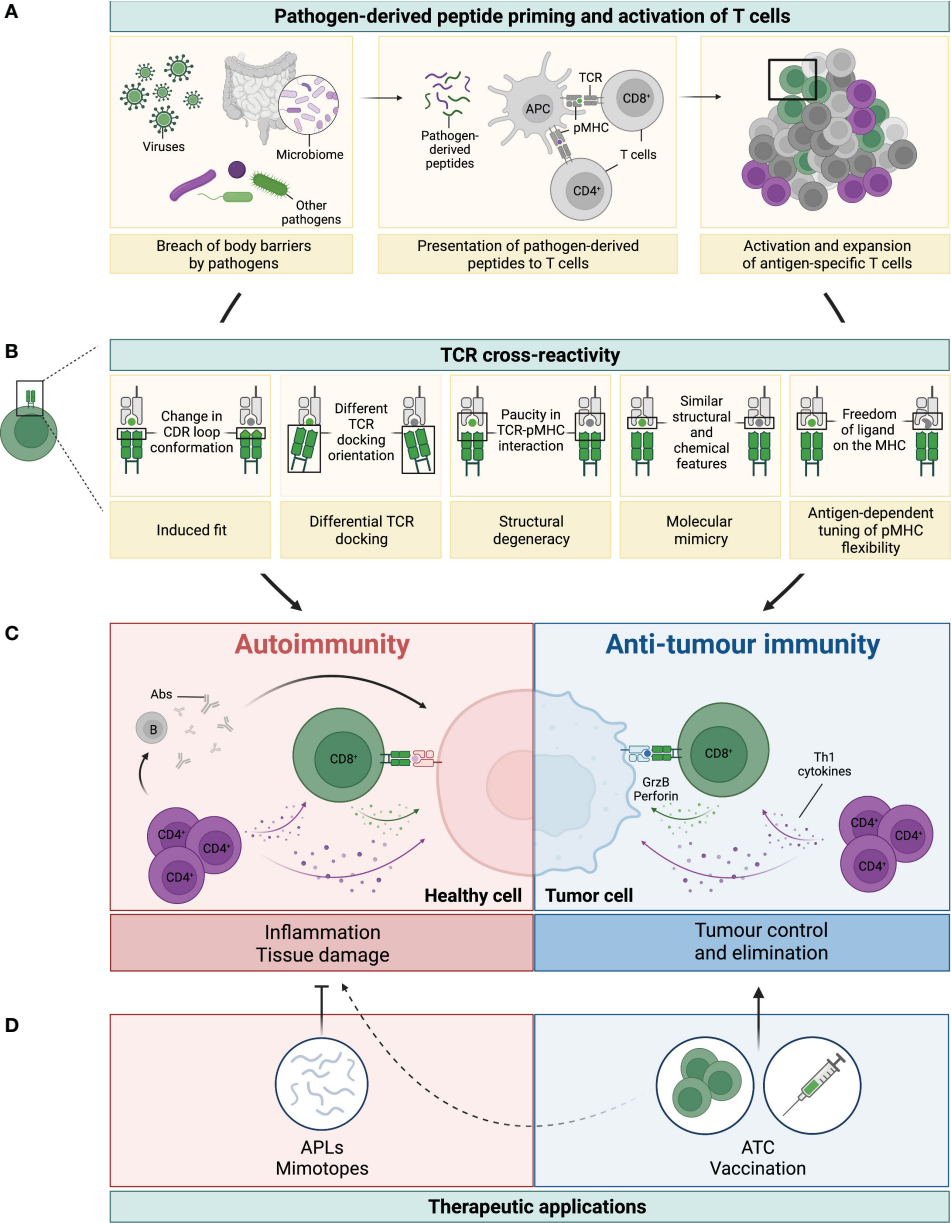
Legend: TCR cross-reactivity: a double-edged sword. **(A)** Microorganisms, such as viruses, microbiota or other pathogens, can penetrate body barriers and get into contact with our immune system (left panel). Processing and presentation of pathogen (foreign)-derived peptides by antigen-presenting cells (APCs) on MHC-class I (represented by the green dot) and MHC-class II (represented by the purple dot) primes and drives the activation of CD8^+^ and CD4^+^ T cells, respectively (middle panel). A polyclonal population of activated T cells then proliferates and expands to fight the invading microorganism (right panel). **(B)** Several mechanisms have been reported to be involved in TCR cross-reactivity. This figure has been adapted from reference (3). A representative CD8^+^ T cell and its TCR is shown in green interacting with the pathogen-derived peptide (in green) presented by an MHC-class I molecule (grey). Due to cross-reactivity, the same TCR can interact with another peptide (shown in grey). **(C)** If this peptide is presented by healthy cells (depicted in pink) or by cancer cells (depicted in blue) this can ultimately result in either autoimmunity or anti-tumour immunity, respectively. On the one hand, recognition of healthy tissues by cross-reactive TCRs (mainly from CD4^+^, but also from CD8^+^ T cells), leads to inflammation and tissue damage with deleterious consequences. Secretion of Th1 cytokines by CD4^+^ T cells (colored in purple) can directly affect healthy cells, but also support the activation of auto-reactive B (grey) and CD8^+^ T (green) cells, which then secrete Abs and cytotoxic molecules (i.e. granzyme B and perforin, illustrated with the green arrow and dots), respectively (left panel in **C**). On the other hand, recognition of tumour antigens by cross-reactive T cells can prompt cell killing and tumour elimination, highlighting the contrasting impact of TCR cross-reactivity in this setting (right panel in **C**). **(D)** TCR cross-reactivity can be exploited for therapeutic applications. Usage of mimotopes or APLs to drive a Th1 to Th2 or regulatory switch in autoimmunity is an attractive strategy to reduce tissue inflammation and its damage. In cancer, usage of cross-reactive TCRs in adoptive T cell therapy (ATC), or of pathogen-derived peptides for vaccination are promising nascent strategies. Potential side effects against healthy tissues of this novel anti-cancer therapies (represented by the dotted line) need to be carefully considered to prevent damage and severe toxicities.

T cells are essential players of the adaptive immunity that are not only responsible for long-term immune memory, but also orchestrate innate and adaptive immune responses. Immature T cells undergo a strict selection in the thymus which leads to the release of mature, largely self-tolerant, T cells. Each of these cells bears several 10.000 copies of a unique kind of T cell receptor (TCR) that results from the assembly of two recombined TCR chains (in most cases α and β) (1, 2). The TCRαβ interacts with antigens presented as peptides by cell membrane-bound molecules of the major histocompatibility complex (pMHC) on the antigen presenting cell (APC) or on the target cell, for example, after pathogen infection (**Figure 1A**). Importantly, the binding of peptides to the various MHC allelic products is subject to specific rules (anchor or preferred residues) (4, 5). The diversity of the TCRαβ T cell repertoire is high, but not unlimited. Based on the V, D and J fragments´ recombination at the two chain loci, the theoretical number of single TCRs is estimated to reach at least 10^15^. In fact, the sum of all different TCRs present in the human blood has been estimated to be much less, in the range of 2.5 x 10^7^ for naïve T cells and approximately 100-fold lower for memory T cells (6–8). The number of potential pathogen- (and tumour-) derived epitopes presented as pMHC throughout life might well exceed this number of T cell clones. It became therefore progressively clear that the clonal selection theory, which proposed that one lymphocyte/receptor is available for each single antigen, needed to be revised, and that cross-reactivity, i.e. the ability of single TCRs to recognise multiple peptide sequences, is a frequent event (9–11).

Cross-reactivity is commonly observed when testing nearly identical peptides which differ only in 1 or 2 amino acids (aa) (for a total length of 8-10 aa for a CD8^+^ T cell epitope presented by MHC-class I). This is physiologically highly relevant for fighting rapidly mutating viruses like HIV, SARS-Cov-2 or dengue viruses (9, 12–14). An interesting example is that of HIV elite controllers who are often HLA-B*5701^+^, an allelic product which, according to *in silico* models, is recognised by T cells with high cross-reactive potential (15). Heterologous immunity, whereby T cells cross-react with different viruses, is also frequently reported and has been reviewed elsewhere (16). In addition, many examples of T cells reacting to very different aa sequences are known (17, 18). Kersh and colleagues claimed that a peptide is recognised as long as it contains a motif for binding to the MHC and one key residue for the TCR (18). This concept was refined by the observation that no single residue was strictly required for recognition, if the available residues allow for a sufficient affinity between the MHC and TCR molecules (19). Thus, peptides not sharing a single residue may productively interact with the same TCR. A similar flexibility was also observed for the length of the MHC-class II peptide, with some CD4^+^ T cells requiring as little as four aa for recognition as long as these optimally fit to the MHC and TCR (20). In contrast, in a more recent study based on a unique experimental approach, Birnbaum et al. tested a set of murine and human CD4^+^ T cell clones and observed that the diversity of the peptide sequences recognised by single TCRs could be smaller than previously thought (21). Still, pluriallelic restriction, as well as alloreactivity have also been experimentally observed (22–24), increasing the number of cross-reactivity scenarios. The structural features that rule cross-recognition have been described in detail (3, 14, 17, 23, 25). They include several mechanisms of conformational adaptation of the TCR and pMHC units (e.g. changes in the TCR docking, displacement of the CDR loop), as summarised in **Figure 1B**. Hence, TCR-pMHC interactions are not rigidly conserved, but rather allow for considerable flexibility within the confines of some general orientation and binding rules. It is also important to note that *in vivo*, T cell cross-reactivity is very likely fine-tuned by the set of co-receptors (inhibitory or activating) and adhesion molecules that the T cell expresses at a given time (26).

A very convincing hint that our T cell immunity is shaped by cross-reactivity was provided in the elegant study of Su et al. (27): a search for HLA-DRB1*0401 restricted CD4^+^ T cells specific for HIV-, CMV- and HSV-derived epitopes in the blood of virus-unexposed healthy donors revealed that although the frequency of such cells was very low (< 10 cells per million), a large fraction (variable between individuals but in average > 50%) were found in the CD45RO^+^ subset. Looking at HIV-specific cells more precisely, the authors confirmed that these CD45RO^+^ cells represent a memory cell pool by assessing IFN-γ production, sequencing the TCR, and analysing further memory markers by gene expression. In addition, such cells were not found in umbilical cord blood. Finally, cross-reactivity of HIV-specific T cell clones with a range of bacterial- or algae-derived peptides suggested that such cells had been primed by unrelated antigens. Also relevant for vaccination, the authors further showed that Influenza-specific clones derived after Flu vaccination were able to recognise related peptidic sequences derived from other microbes. Similar observations were done by the group of F. Sallusto that demonstrated that HIV-specific CD4^+^ T cells could be detected in both the naive and memory T cell subsets (defined with the two markers CD45RA and CCR7) of HIV-unexposed healthy donors (28). The large majority (>80%) of the HIV-epitopes activating memory T cells matched strongly with human microbiome aa sequences. A further notable observation in this report was that both the specificity and the frequency of these HIV-specific T cells were different across donors. This highlights the inter-individual variability of T cell responses, likely to be shaped by both MHC-polymorphism and the environment.

Despite clear evidences about the cross-reactive nature of the TCR, it remains unclear how many single peptides can a unique TCR recognise in “real life”. According to early estimates, it should be approx. 10^6^ (29). Meanwhile, there is evidence from several studies that individual T cell clones can indeed sense over a million different peptides in the context of a single MHC molecule (30–32). Studying the cross-reactive repertoire of an autoimmune HLA-A*0201 CD8^+^ T cell clone which recognises a 10 aa-long preproinsulin-derived peptide, Sewell and co-workers showed that many of the “cross-reactive” peptides were better agonists than the original one, despite some sequences differing in up to 7 out of the 10 aa positions (31). Based on the assumption that only 1% of all peptides will end up being presented on MHC, they also estimated the “true” frequency of cross-reactivity to be approximately 1 in 10^4^ peptides (11, 31). This is in the same range as the frequency of 1 in 3x10^4^ found by Ishizuka et al. when using a peptide library derived from pathogen sequences (33).

## 2 T cell cross-reactivity and autoimmunity

The most obvious and detrimental consequence of T cell cross-reactivity to vast numbers of individual peptides is the risk of developing autoimmunity (**Figure 1C**, left panel). Although self-reactive T cells are deleted in the thymus, weakly cross-reactive T cells may survive and become activated in the periphery through the recognition of epitopes from infectious agents (microorganism antigens, MoAs), a phenomenon known as “molecular mimicry”. Memory T cells can be stimulated by peptide concentrations more than 50-fold lower than those required to stimulate naïve T cells (34, 35). It is, therefore, likely that a memory T cell could be stimulated by a cross-reactive self-peptide with an affinity for the TCR that is far lower than that of the original pathogen-derived peptide. This goes in line with the quite frequent observation that infections can precipitate autoimmune diseases (36), and is of particular interest for novel therapies (37, 38). In autoimmunity, preferentially TCR cross-reactivity of CD4^+^ T cells has been analysed as a consequence of their central role in the development of autoimmune disorders. This is in contrast to cancer where analysis of cytotoxic anti-tumour response, i.e. CD8^+^ T cells is more important.

Here, we mainly present three examples for the involvement of TCR cross-reactivity in the induction of autoimmune diseases: one resulting from a bacterial infection, i.e. rheumatic fever; another induced by a food component, i.e. celiac disease; and a third example representative for the many autoimmune disorders for which no clear connection to an environmental agent has been found, as for instance multiple sclerosis.

### 2.1 Rheumatic fever (RF)

Acute rheumatic fever is a typical example of systemic autoimmunity which occurs subsequently to an infection, namely with group A β-haemolytic streptococci (39, 40). It can affect synovial joints, cardiac valves and the brain, resulting in clinical features as arthritis, carditis, chorea, erythema marginatum and subcutaneous nodules. Molecular mimicry between group A streptococci and heart tissue was first described by Kaplan in 1960 (41). In the early 1980s, the role of both humoral and cellular autoimmune responses was reported in several studies (42). The cross-reactive antibody (Ab) response against *S. pyogenes* has been well described (43, 44). Meanwhile, it is clear that also T cell-mediated immune reactions play an important role in RF (40, 44, 45). Three types of protein antigens present on the *S. pyogenes* surface are M, T, and R proteins. M protein is the most virulent one and shares structural similarities with various host proteins, including cardiac myosin, laminin, vimentin, and tropomyosin (43, 46, 47). During this cellular response, streptococcal antigens are presented via MHC-class II molecules and activate autoreactive T cells (40). Indeed, T cells from patients with RF recognise different alpha (α)-helical coiled-coil proteins such as streptococcal M protein, myosin, laminin, and tropomyosin, and identical epitopes on the N-terminal portions of both streptococcal M protein and cardiac myosin were identified (45). In addition, in the valvular tissue and myocardium of patients with RF, T cells with three patterns of cross-reactivity were found: 1) cardiac myosin and valve-derived proteins, 2) cardiac myosin and streptococcal M peptides, and 3) cardiac myosin, streptococcal M peptides and valve-derived proteins (48). Potential sites of mimicry were revealed in the S2- and light meromyosin (LMM)-region of human cardiac myosin peptides and distinct peptides in the B repeat region of streptococcal M protein (peptides B2 and B3A) (45). Other mechanisms which are involved in the pathogenesis of RF are epitope spreading and TCR degeneracy. Ellis et al. investigated the degeneracy of the cross-reactive T cell responses towards different α-helical proteins such as human cardiac myosin, laminin, tropomyosin, and streptococcal M protein, and observed a mosaic of different T cell clones reacting with at least six distinct α-helical proteins demonstrating different degrees of cross-reactivity (45). Moreover, T cells are activated in RF when auto-Abs interact with the endothelium cells, leading to upregulation of vascular cell adhesion molecule 1 (VCAM-1) and facilitating increased T cell infiltration into the heart valve (49). These activated auto-reactive T cells produce inflammatory cytokines and lead to valve damage but also promote activation of B cells which produce cross-reactive Abs. Due to the destruction of valvular tissue, epitope spreading may occur, thus enhancing the humoral and cellular autoimmune reaction.

Another crucial streptococcal antigen is N-acetyl ß D-glucosamine (GlcNac), a carbohydrate moiety of the bacteria cell wall (43). In a neurologic manifestation of RF, the Sydenham chorea, T cells as well as Abs that recognise this bacterial antigen have been shown to cross-react with the brain cell antigens lysogangliosides and tubulin (39, 50, 51). The humoral responses correlate with clinical symptoms and mediate neuronal cell signalling (52).

### 2.2 Celiac disease (CeD)

Celiac disease is highly interesting in view of the fact that autoimmune reactions are induced by a food component, i.e. dietary gluten (gliadin in wheat, hordein in barley, and secalin in rye are the most prominent examples). Antibodies against gliadin-peptides and the enzyme transglutaminase-2 (TG2) are highly-specific diagnostic markers of CeD, and a CD4^+^ T cell response towards post-translationally modified gluten peptides has been described. The disease shows a clear genetic association to the MHC-class II allelic products HLA-DQ2 (DQ2.5: DQA1*05:01-DQB1*02:01 or DQ2.2: DQA1*02:01-DQB1*02:02, approx. 95% of the patients) and HLA-DQ8 (DQA1*03:01-DQB1*03:02, approx. 5% of the patients) (53).

Interestingly, gliadin is a substrate for the TG2 enzyme which catalyses deamination at glutamine residues. The conversion of Q to E aa leads to increased binding affinity of peptides to the HLA-DQ2.5/2.2/8 molecules and enhanced recognition by gluten-specific CD4^+^ T cells (54–56). Hence, CeD-associated T cells preferably react with “self-produced mimotopes” that result from the deamidation of gliadin-derived peptides. Another level of cross-reactivity that has been documented in CeD is the recognition by a single DQ2.5-restricted TCR of peptides of similar, but not identical, aa sequences derived from various gliadins (i.e. α1a and ω1) (57). To which extend this cross-reactivity participates in the immune response against various gliadins and/or hordein or secalin is still not fully investigated, but is starting to be explored at large-scale (57–59). Altogether, the strong anti-gluten CD4^+^ T cell response present in CeD is providing help to B cells that bind TG2-gliadin complexes and deaminated gluten peptides to mature into plasma cells in the gut that in turn produce deaminated gluten-specific, as well as autoreactive, TG2-specific, Abs (60–62). In addition, gluten-specific CD4^+^ T cells are consistently found in the small intestine of celiac disease patients, where they activate intraepithelial CD8^+^ T cells (IELs) via the production of IFN-γ, IL-21 and IL-2 (62). Although these IELs are thought to largely contribute to disease pathogenesis, the link between the gliadin-specific CD4^+^ T cell response and the recruitment and activation of IELs in the gut remains obscure, especially because these IELs have not been shown to recognise gluten.

Even if there is ample evidence that HLA-DQ2.5, HLA-DQ2.2, or HLA-DQ8 molecules present gluten-derived peptides, expression of these allelic products alone is insufficient to cause disease. Other risk factors which may induce increased expression and activity of TG2 may also be involved. For instance, *in vivo* and *in vitro* studies support an association between gut microbiota alterations and celiac disease (63). First, the microbiota composition differs between individuals with active celiac disease, patients on a gluten-free diet, and normal controls in both oral, duodenal and faecal samples, with an increase in virulent strains noted in patients with active CeD (64). Bacteria can modify immunogenic food antigens resulting in an increase or decrease in antigenicity, and also utilise undigested particles as substrates, producing metabolites such as short-chain fatty acids that affect intestinal homeostasis. For instance, *Pseudomonas aeruginosa*, an opportunistic pathogen isolated from CeD patients, processes gluten to T cell reactive epitopes whereas bacterial species from healthy controls inactivate these reactive epitopes by further proteolytic breakdown (65). Second, and more relevant in the context of T cell cross-reactivity, peptides from common commensal and pathogenic bacteria, especially from several *Pseudomonas* and *Bordetella* species can mimic gliadin-derived peptides and activate gliadin-specific, HLA-DQ2.5-restricted T cells from CeD patients (66). It has been, therefore, hypothesised that celiac disease may be induced not only by gluten ingestion but also by infectious processes inducing pathogen-specific T cells that cross-react with gluten epitopes (66).

### 2.3 Multiple sclerosis (MS)

Multiple sclerosis is one of the most prevalent autoimmune disorders of the central nervous system (CNS), and is characterised by the loss of the protective myelin sheath that surrounds the axons of neurons (67, 68). Its pathophysiology has been extensively studied, especially in experimental allergic encephalomyelitis (EAE) which is a generally accepted animal model for the human disease. Nevertheless, the aetiology of MS is still unclear. Its association with an infection has been postulated already in the late 1800s, after it was first described (67). Nowadays, several factors such as genetic susceptibility, environment including infectious agents, obesity, lack of sun exposure and vitamin, have been suggested to be involved (69).

Autoantibodies specific for a variety of CNS proteins, as for instance myelin basic protein (MBP) or myelin oligodendrocyte glycoprotein (MOG), are present in the serum, cerebrospinal fluid (CSF), and brain of MS patients (70). Similarly, CD4^+^ T cells specific for myelin antigens are found in the blood. Studies on antigen recognition demonstrated that CD4^+^ autoreactive, MBP-specific, T cells from MS patients cross-react with peptides derived from bacterial or viral proteins (71, 72). As shown by structural analyses performed by Lang et al., the same TCR binds a MBP peptide presented by HLA-DRB1*1501 and an unrelated Epstein Barr virus (EBV)-derived peptide bound to HLA-DRB5*0101, a typical example of molecular mimicry (73, 74). A link between EBV infection and MS had already been suggested by the observation that the infection may precede MS pathology and the identification of cross-reactive Abs in MS patients (67). EBV is a well-investigated candidate for antigenic mimicry, from mimotope peptides recognised by T cells to cross-reactive Abs (75). Also, an altered anti-EBV T cell reaction was suggested in MS (76, 77).

These findings led to the concept that an immune response initially activated and expanded by an infectious agent may, in general, cross-react with autoantigens mediating CNS inflammation and induce destruction of the brain. To date, numerous infectious agents have been described to induce cross-reactive T cells against brain-specific epitopes. As an example, peptides from HSV and *Pseudomonas aeruginosa* bound to MHC molecules are recognised by cross reactive myelin-specific T cells (78). Furthermore, peptides from *M. tuberculosis*, *S. typhimurium* and *E. coli* lead to strong *in vitro* proliferation of MBP-specific T cells and induced EAE in mice with the same severity and incidence as the autoantigen peptide of MBP (79).

T cell clones isolated from the blood of patients with MS show high specificity for the immunodominant MBP epitope MBP_85–99_ (80). However, this specificity is not absolute. Indeed, changing the TCR contact residue lysine at position 93 to an arginine, or even just removing a hydroxyl group by changing a phenylalanine to a tyrosine at position 91, can totally abrogate T cell reactivity. This lysine-to-arginine substitution can also result in a more degenerate pattern of TCR recognition, in that a tyrosine or other aa residues can now be tolerated at positions 91 or even 90 (81). Hence, while a TCR appears to be highly specific in one situation, altering the peptide ligand can change the TCR conformation to yield a higher degree of T cell cross-reactivity. Analysis of a further series of MBP_85–99_ reactive T cell clones led to a similar conclusion, showing that a number of virus-derived epitopes can trigger autoreactive T cell clones in a manner that would not be predicted by simple algorithms (71). One of the studied MBP-reactive T cell clones recognised an epitope of MOG, an entirely different self-protein. Thus, a significant degree of functional degeneracy exists in the recognition of self-antigens by T cells.

### 2.4 Evidence for TCR cross-reactivity in other autoimmune diseases

The link between infection and autoimmunity *via* molecular mimicry has also been investigated in other inflammatory CNS diseases, particularly in chronic Lyme disease. Following acute infection with *Borrelia burgdorferi* (Bb), a chronic inflammatory disease can emerge which targets joints or the CNS in the absence of residual bacterial infection. In this condition, an autoimmune response to self-antigens (similarly as described above for RF) may arise from bacterial-specific T cells (82). Indeed, in Lyme arthritis, CD4^+^ T cells isolated from the synovial fluid of patients were shown to recognise a 9mer peptide from an outer surface antigen from Bb (OspA_165–173_) and an analogous, but not identical, sequence from the human LFA-1 molecule (CD11a_332–340_) (83). Similarly, Bb-specific T cells from the CSF of a patient with CNS manifestation of borreliosis cross-reacted with several self-antigens, one of them being a myelin antigen (84).

In uveitis, it has also been shown that peptides with similar structure rather than similar aa sequences can induce cross-reactive T cell responses. For instance, similarities of 6 to 7 aa with the 14mer autoantigen peptide from retinal S-antigen (PDSAg) with peptides of 11 or 12 aa in length from different environmental proteins is sufficient to induce autoreactive CD4^+^ T cell recognition and experimental anterior uveitis in rats (85). Although the pathogenic cells in uveitis are MHC-class II restricted CD4^+^ T lymphocytes, statistical associations with HLA-class I molecules (B*27, B*51) are well known. Interestingly, the HLA-class I molecule seems to serve as an autoantigen itself, being presented as a peptide (B27_125–138,_ termed B27PD) on HLA-class II and mimicking the retinal PDSAg peptide (86). Oral administration of B27BP peptide to patients was also shown to improve uveitis symptoms, suggesting that cross-reactivity could be even exploited for inducing oral tolerance to autoimmune antigens (87). In a recent study including patients with acute anterior uveitis and ankylosing spondylitis, an HLA-B27-linked rheumatic disease frequently associated with uveitis, TCRs responding to HLA-B*27-bound peptides derived from microbial antigens or from self-antigens were identified. These peptides shared common TCR binding motifs, supporting the idea that HLA-B*27-presented microbial peptides could act as trigger for autoimmunity by activating anti-self CD8^+^ T cells (88). Interestingly, the ankylosing spondylitis-associated TCRs showed weaker affinity for the human peptide ligands than for a peptide from a conserved bacterial inner membrane protein. Evaluating the structures of seven of the HLA-B*27:05 peptide-TCR complexes, the authors showed that in all of these structures, the TCRs used a similar solution to interact with the conserved motifs in the self and bacterial peptides (89).

In type I diabetes, a T cell-mediated, HLA-DQ2 (DQA1*05:01-DQB1*02:01) and -DQ8 (DQA1*03:01-DQB1*03:02)-associated autoimmune disease directed at pancreatic β cells, insulin B-chain_9-23_ (B:9-23) is a key epitope presented by MHC-class II to CD4^+^ T cells targeting pancreatic β-cells. Lack of an acidic aa residue (i.e. aspartic acid and glutamic acid) at position 57 of the DQ8 β chain of the MHC molecule favours binding of the insulin-B peptide and is associated with increased risk of developing the disease (90). Without this acidic residue, the presented peptide repertoire is typically negatively charged (91, 92). Mimotopes with acidic aa substitutions at P9 have been shown to detect self-reactive, IFNγ-producing T cells much stronger than the wild-type peptide (93, 94). Interestingly, an immune response to this mimotope was also observed in control subjects without diabetes, but in these individuals, rather IL-10 producing, hence, anti-inflammatory CD4^+^ T cells were activated (93).

### 2.5 Therapeutic implications in autoimmune diseases

Distinct cytokine patterns of T cell subsets make them unique and define their role in host defence or their contribution in disease pathogenesis. In autoimmune diseases, the role of Th1 and Th2 cells along with their cytokine profiles is well documented. In particular, the priming signal (specificity, affinity and avidity of the pMHC/TCR, APC/T cell interaction) controls the maturation, differentiation and function (i.e. cytokine profile) of the T cell (37). Alteration of peptides and of their binding to MHC may, therefore, influence the strength of the immune response. For the development of therapeutic agents in autoimmune diseases, silencing the armful anti-self T cell activity by either shifting the inflammatory Th1 response towards a Th2 profile, inducing regulatory T cells (Tregs), or even completely inhibiting T cells using strong antagonists are all strategies of interest (**Figure 1D**, left panel). Many of such “mimotopes” have been meanwhile designed based on *in vitro* testing of the responsiveness of T cells isolated from patients or *in vivo* using animal models (38). Especially those reducing pathogenic responses have been tested for therapeutic purposes in clinical trials.

The ability of altered peptide ligands (modified peptide sequences derived from an original antigenic peptide, i.e APLs) to shift an unfavourable Th1- in a more favourable Th2-response in the murine EAE model of MS has first been shown by Nicholson and colleagues (95). The authors used an analogue of the encephalitogenic myelin proteolipid PLP_139-151_ (the common T cell antigen in EAE) with substitutions at the two main TCR contact residues (L144/R147) which had been shown to be a powerful TCR antagonist for the encephalitogenic PLP-specific T cell clones *in vitro* (96). Injection of this analogue protected the animals from developing EAE. Kuchroo et al. showed that this APL can activate IL-4 secretion by both encephalitogenic T cells and naive T cell clones that cross-react with self-antigens and inhibit autoimmunity by the induction of Tregs leading to bystander suppression of EAE (96). In further animal models of EAE, APLs have been proven to have a significant therapeutic value (97). Meanwhile, autoreactive human T cell clones have been shown to secrete the anti-inflammatory cytokines IL-4 and TGF-β after TCR engagement by APLs (98). However, application of APLs in MS may be a double-edged sword. On the one hand, it was shown that an altered MBP_85–99_ peptide induces Th2 cytokine secretion by MBP-reactive T cells isolated from the peripheral blood of MS patients while on the other hand, it can induce disease in some patients by activating these MBP-reactive T cells against the patient’s own tissues (99). Moreover, in a phase II clinical trial with this peptide, two out of seven MS patients developed high frequencies of MBP-reactive T cells, and these responses were associated with significant increases in MRI-detectable lesions (100). In contrast, patients treated with lower doses of the same APL experienced some degree of immune deviation towards increases in IL-4 secretion by MBP-reactive T cells (101, 102).

A mimotope was also developed for patients with diabetes mellitus type 1 in order to preserve pancreatic β cell function. It was modified from the human insulin peptide B:9-23 which binds to HLA-DQ8 and is recognised by CD4^+^ T cells present in the islets of organ donors with type 1 diabetes ((103) and section 2.4). The substitutions in this modified peptide are known to be important in the diabetes-prone NOD mouse model (104, 105). However, a four-arm phase II clinical study conducted by Walter et al. could not show any clinical improvement (as measured by C-peptide concentrations, a measure of pancreatic β cell function), after subcutaneous administration of the mimotopes over two years compared to the placebo (103).

For celiac disease, and as mentioned in section 2.2, disease-associated T cells preferably react with “naturally produced mimotopes” that result from deamidation of gliadin-derived peptides. Epitope-specific immunotherapies are, therefore, a logical translational step. In HLA-DQ2.5-positive celiac disease patients, clinical trials using a combination of three gluten-derived peptides, which contain at least five gliadin-specific T cell epitopes presented by HLA-DQ2.5 (Nexvax2) were conducted. While the phase I studies showed preferable outcomes in terms of safety and tolerability, the recent Nexvax2 phase II trial had to be discontinued due to lack of protection to gluten challenge.

An alternative approach to the use of a single APL is the administration of peptide mixtures that contain many different antigen specificities. Random copolymers that contain aa commonly used as MHC anchors and TCR contact residues have been proposed as possible “universal APLs.” The synthetic immuno-active copolymer glatiramer acetate (GA) is comprised of four aa in random order with an average length of 40-100 residues which resemble MBP (106). It was first synthesised in 1967 to induce EAE in murine models, but was then unexpectedly found to reduce signs and progression of the disease (107). Rather than inducing an autoimmune disease, GA was found to induce regulatory and protective neuroimmune responses. In most patients, daily injection with GA causes a striking loss of responsiveness to this polymer antigen, accompanied by greater secretion of IL-5 and IL-13 by CD4^+^ T cells, indicating a shift towards a Th2 response (108, 109). In addition, the GA-reactive T cells exhibit a high degree of degeneracy, as measured by their ability to cross-react with a large variety of peptides represented in a combinatorial library (108). GA-induced migration of those highly cross-reactive Th2 (and perhaps regulatory FoxP3^-^ Th3) cells to the sites of inflammation may allow their highly degenerate TCRs to contact self-antigens, which they recognise as weak agonists. These T cells then apparently secrete suppressive, Th2/Th3 cytokines, thus restricting local inflammation (108). Due to these beneficial effects, GA was approved for therapeutic use in 1996 and is since then a first-line treatment of relapsing remitting MS (110, 111).

## 3 TCR cross-reactivity in the context of cancer

With the notable exception of rare antigenic aberrant sequences, e.g. mutated antigens, tumours generally present self-antigens on their MHC molecules and are poorly immunogenic (112). This can be globally seen as the result of the thymic negative selection where highly self-reactive T cells are eliminated to prevent the development of autoimmune diseases, leaving us with a TCR repertoire with only low to moderate affinity to self-antigens (113). Although this is beneficial in a healthy state, it makes tumour targeting by T cells a hard task, as it impairs the mounting of an effective and strong immune response. Hence, in contrast to the situation in autoimmune diseases, cross-reactivity of potential pathogen-specific T cells against self-antigens specifically presented by tumour cells is not only desirable, but would likely result in favourable anti-tumour immunity (114).

An early and staggering example of TCR cross-reactivity was described by the group of P. Romero for the tumour-associated antigen (TAA) Melan-A. While the frequency of any antigen-reactive T cell in the peripheral immune naïve repertoire is generally extremely low (< 1 in 100.000 T cells), up to 1 out of 1000 CD8^+^ T cells bind the immunodominant peptide from Melan-A_26-35_ (the modified A27L ligand), when presented by HLA-A*0201, both in healthy donors as in melanoma patients (115). Although numerous T cells were able to bind the pMHC, as assessed by MHC-tetramer staining, a subgroup failed to be significantly activated by the Melan-A peptide in a cytotoxicity assay. In contrast, several other tested peptides, which included proteins of self- or pathogen- origin, generated a strong response in the same assay, hinting at the highly cross-reactive nature of this repertoire of T cells (116). Further supporting this, a following study of the same group showed that a tumour-reactive CD8^+^ T cell clone, also specific to the same immunodominant peptide mentioned above, was able to cross-recognise numerous peptides and that stimulation of this clone with these peptides drove the expansion of a heterogeneous CD8^+^ T cell population, with only a fraction actually reacting to the Melan-A peptide (117). Importantly, immunisation with Melan-A peptide through vaccination leads to a reduction on the population of cross-reactive T cells and an enrichment of antigen-restricted T cells that can react with the tumour (118). These early works on TCR cross-reactivity demonstrated its relevance not only in tumour biology but also in the design of effective anti-cancer immunotherapies.

### 3.1 Evidence for tumour antigen recognition by pathogen-specific T cells

#### 3.1.1 T cell cross-reactivity between virus-derived sequences and tumour antigens

Studies have described viral-specific T cells within the microenvironment of several tumour entities with no prior known viral aetiology (119). Although there is experimental evidence for the presence of intracellular bacteria or viruses in tumour cells (120–122), this local pathogen load might not be the only reason for the presence of pathogen-specific T cells within tumours. After sequencing the TCRs of tumour-infiltrating lymphocytes (TILs) in non-small cell lung carcinoma (NSCLC), Chiou et al. identified a novel TAA derived from the epithelial protein TMEM161A. A TCR recognising this peptide was shown to readily cross-react with epitopes from EBV (and *E. coli*). Specific T cells were not only found in NSCLC patients, but also in healthy donors, an observation which the authors offer as an explanation to the presence of virus-specific T cells within NSCLCs, but possibly also in other tumours (123). In another *in silico*-based approach, Ragone et al. examined the cancer peptide database and identified numerous TAAs with shared homology with viral sequences. The viruses whose sequences were most commonly shared with the tumour antigens were HIV type 1 (HIV-1), HSV, and human papillomaviruses (HPV). In addition to sequence homology, the authors also report that these peptides share structural similarities with comparable patterns of contact between the HLA molecule and the TCR (114). A recent case report has also described tumour reduction in three metastatic colorectal cancer patients upon SARS-CoV-2 infection (124). Altogether, these studies point out to the fact that pathogen- and tumour antigen- cross-reactive T cell responses might play an important role in anti-cancer immunity, and that the immune repertoire of each patient, shaped by previous infections, might be a crucial factor in disease control.

In murine melanoma models, Chiaro et al. showed that similarities between tumour- and viral- derived antigens can influence the clearance of tumours upon peptide cancer vaccination as a consequence of cross-reactive T cell activity. Upon immunisation with viral peptide pools previously selected based on their homology to tyrosinase related protein (TRP2_180–188_) or glycoprotein 100 (gp100_25–33_), a strong reduction in tumour growth was seen. Interestingly, the authors further argue that viral molecular mimicry is an important factor that dictates immune response also in metastatic human melanoma by showing a direct correlation between pre-existing Abs against CMV, and response to the immune checkpoint inhibitor (ICI) anti-PD-1 (125). TCRβ sequencing experiments further suggested that the same T cell clone recognised similar peptide sequences of MAGE-A10 and CMV. Further studies using pre-clinical murine models suggest the relevance of activating virus-specific T cells for tumour growth control (119, 126). The authors describe the formation of an immune-permissive microenvironment upon *in vivo* virus-peptide vaccination, whereby cross-reactivity of these viral-specific T cells with tumour antigens, although not tested, could be responsible for the effect observed. Interestingly, another study simulating immunisation of mice with the TAA and homologous viral peptides predicted a similar clearance of tumour cells in both scenarios, suggesting equivalent anti-tumour efficacy of the effector T cell response (114).

#### 3.1.2 T cell cross-reactivity between bacterial-derived sequences and tumour antigens

Cross-reactivity of tumour-specific T cells with bacterial epitopes has also been described. In melanoma, a MAGE-A6-derived peptide (MAGE-A6_172-187_) was shown to be cross-reactive with its highly immunogenic homolog HF-2_216-229_. This mycoplasma-derived peptide and MAGE-A6 can drive the formation of memory CD8^+^ T cells. Interestingly, *in vitro* priming with dendritic cells loaded with the bacterial-derived peptide resulted in CD8^+^ T cells with 100-fold higher avidity to the MAGE-A6 peptide compared to that of cells primed with the MAGE-A6 peptide itself (127).

The main *in vivo* source of bacteria-derived antigens is the microbiota. The human gut is colonised by approximately 10^14^ microbes (128). The sheer number of colonising microorganisms means that exposure of immune cells to these bacteria throughout life is unavoidable, which results in the generation of an immune response against commensal-derived peptides. In a similar analysis to the one performed earlier, Ragone et al. compared all TAAs from the cancer peptide database against the microbiota species *Firmicutes* (taxid:1239) and *Bacteroidetes* (taxid:976) sequences. The authors demonstrated a high level of homology of tumour antigens and peptides derived from these species, which account for 90% of all gut microbiota (129). Flückiger et al. showed that T cell clones that recognise the cancer antigen protein glycerol-3-phosphate dehydrogenase 1-like (GPD1-L) and cross-react with epitopes derived from the tail tape measure protein (TMP) of an *Enterococcus hirae* (*E. hirae*) bacteriophage, could be detected in melanoma patients. Importantly, the authors further observed an association between the presence of this prophage in the stools of patients with renal and lung cancer, expression of GPD1-L by tumour cells, and a long-term benefit to PD-1 checkpoint blockade (130). Interestingly, in the same study, cyclophosphamide treatment of tumour-bearing mice, which induces the translocation of *E. hirae* from the gut lumen to the mesenteric and splenic immune tissues, resulted in improved anti-cancer CD8^+^ T cell responses. This anti-tumour effect was abrogated once the mice were given antibiotics and rescued by administration of *E. hirae* isolates. Moreover, lack of expression of the TAA by the tumour cells also abolished any anti-tumour immunity previously observed.

Other studies have also shown a favourable clinical outcome in cancer patients presenting CD4^+^ and CD8^+^ T cells specific for *E. hirae*, *Bacteroides fragilis*, *Ruminococcaceae* (131), and *Akkermansia muciniphila* (131–134). The immune repertoire, namely the frequency of precursor T cells prior to antigen exposure, is a critical factor in determining the magnitude of an immune response. Based on the aforementioned observations of cross-reactivity between numerous pathogen-derived epitopes and tumour antigens, it is plausible that the gut microbiome is an important modulator and dictator of how individuals will mount an immune response to tumours but also how they will respond to immunotherapies.

### 3.2 Neoantigens and T cell cross-reactivity

In contrast to the demonstrated potential of T cells to be cross-reactive (11), neoantigens generally activate specific T cells that react only very weakly against the wild-type (wt) peptide which often differs only in 1 aa (135–138). This apparent contradiction may be explained when considering the position of the mutated aa in the peptide sequence (e.g. if a novel anchor residue for binding to the MHC molecule is created by the new aa) or its structural properties (e.g. changes in peptide charge which renders the peptide “visible” to the TCR). Still, the large majority of predicted neoantigens probably activate similar TCRs to that specific for the self-peptide and are, therefore, not of interest. If this is the case, these neoantigens do not trigger a strong anti-tumour response as a result of central tolerance. On the other hand, neoantigens can share homology to pathogen-derived antigens. In this case, these neoantigens could elicit an efficient response against tumours by activating cross-reactive pre-existing memory T cells that have been previously generated against such pathogens, as discussed above for wt tumour antigens (139). Bessel et al. identified an epitope (SVYRYYGL (SVY)) derived from the genome of the commensal *Bifidobacterium breve* (*B. breve*), homologous to the neoepitope expressed by the murine model B16-SIY (SIYRYYGL (SIY)) (140). They further demonstrate that *B. breve* promotes the expansion of SVY-specific CD8^+^ T cells and that these are able of effective tumour control in SIY-expressing tumours, although comparison with SIY-specific T cells was not performed. In pancreatic cancer patients, Balachandran et al. demonstrate that the quality of the tumour neoantigens, namely the similarity to pathogen-derived epitopes, rather than the quantity, greatly associates with long-term survival (141).

Importantly, cross-reactive neoantigens seem to be a critical predictive factor for checkpoint inhibitor therapy efficacy. In the seminal study by Snyder et al. which first identified mutated antigens as T cell targets during checkpoint blockade, the authors observed that patients with long-term benefit to anti-CTLA-4 therapy share neoepitopes homologous to more viral and bacterial antigens, in contrast to patients with minimal or no benefit (142). These intriguing findings strongly suggest that cross-reactive T cells specific for pathogens can get activated upon checkpoint inhibition and participate in a clinically significant anti-tumour response. This is in line with the different studies presented above where the importance of the gut microbiome in checkpoint therapy responsiveness has been highlighted (143).

### 3.3 The two faces of TCR cross-reactivity in tumour immunotherapy

In addition to being able to dictate the outcome of immunotherapies such as checkpoint inhibition and therapeutic cancer vaccination with tumour-derived antigens, TCR cross-reactivity is currently being exploited for the development of novel and more potent cancer therapies, which we will discuss in more detail below.

#### 3.3.1 Overcoming self-tolerance

##### 3.3.1.1 Improving affinity

If numerous T cell clones recognise the same epitope, affinity and avidity for this epitope will be inevitable highly variable. Using checkpoint inhibitors will unleash the inhibition in all lymphocytes present in the tumour microenvironment (TME), high or low functional ones. Differently, the goal of therapeutic vaccination is to selectively drive the recruitment of high-avidity T cells and promote strong and long-lasting anti-tumour responses. As mentioned above, high-affinity T cells against TAAs are usually lacking as a consequence of negative selection in the thymus, which leaves us only with a low-affinity repertoire. This tolerance is observed when A2xneu mice (Her2/neu mice crossed with A2.1/K^b^ mice) are injected with the immunodominant Her2_773-782_ peptide, which results in little to no tumour control (144). A similar tolerance was observed when mice were injected with p53-derived peptides. In this case, the authors demonstrated that using the p53_261-269_ self-epitope led to the expansion of cytotoxic T lymphocytes (CTLs) in p53 wt mice with an avidity more than 10-fold lower than the ones obtained from p53 null mice (145). This nicely shows the importance of circumventing tolerance to achieve an effective cancer vaccination.

One way to improve the immunogenicity of TAAs would be to exploit the cross-reactive nature of TCRs. Identification of peptides that are not naturally processed and presented but that can be used to elicit strong cross-reactive T cell responses against the original TAAs is already an old idea. The design of such heteroclitic peptides, where the stability of interaction between the peptide and MHC molecule is improved by replacement of certain aa was shown to be a powerful strategy for both improving CTL reactivity *in vitro* and controlling tumor growth in mice (144, 146–150). Importantly, these heteroclitic peptides need to be recognised by T cells that cross-react with the native sequence and can, therefore, drive the killing of tumour cells naturally presenting the original peptide. Despite the encouraging results seen in pre-clinical models, this concept has failed yet to lead to the development of an effective cancer therapeutic vaccine (151, 152). A famous example was the observation by Speiser et al. that immunisation of melanoma patients with the wt Melan-A_26-35_ (together with CpG as adjuvant) was superior in generating high avidity, tumour-reactive T cells, compared to the Melan-A_26-35_ modified peptide (152). Since the only difference between the two peptides is one aa substitution at an anchoring position (A27L), it suggests that increasing pMHC binding properties is not the ultimate key for improving T cell reactivity to TAAs.

##### 3.3.1.2 Microorganism antigens (MoAs) molecular mimicry

Recently, a novel concept exploiting TCR cross-reactivity for therapeutic purposes has emerged. It is based on the identification of natural analogue peptides capable of inducing strong T cell responses against the tumour antigen. The shared homology between pathogen-derived peptides and tumour antigens and the aforementioned correlations between cross-reactive T cells and clinical outcome makes this an attractive and promising strategy that is currently being further investigated.

We have introduced in sections 3.1 and 3.2 that tumour antigens share homology with numerous pathogen-derived epitopes which, as a consequence, can drive the activation of T cells that share the same TCR. In other words, T cells that have been activated upon exposure to a certain pathogen can cross-react with tumour antigens (**Figures 1A–C**). The reasons for exploiting this cross-reactivity in the context of therapeutic cancer vaccination are manifold: first, it allows to overcome the low immunogenicity and affinity of natural TAAs, since TCRs that recognise MoAs have not been depleted from the T cell repertoire. Second, memory T cells can be activated by much lower peptide concentrations as compared to their naïve counterparts (see section 2). Third, recalling T cell responses upon immunisation is obviously easier to achieve than priming new effectors, especially when considering the current lack of gold-standard strong adjuvants. Fourth, exploiting “natural” T cells that were already expanded in the body after infection should present less risk of autoimmunity, although, as exemplified in section 2, autoimmunity cannot be fully excluded.

In summary, activation of viral- or commensal- specific T cells that cross-react to the tumour cells have shown promising results in a couple of pre-clinical models. Furthermore, correlations between the presence of these T cells and clinical outcome in patients have also been drawn. All this is opening a new field of research, to identify tumour antigens and MoAs that share high homology for the developing of novel T cell-based immunotherapies for cancer (**Figure 1D**). Since the presence of MoAs-specific memory T cells depends on prior infections, the composition of the microbiota, and the MHC-allotype, one could speculate that the development of such strategies should be done in an individualised manner to guarantee a high success rate and decrease the risk of side effects. Combination of such therapeutic vaccinations with ICIs could unleash the expansion of potent effector memory cells that readily target the tumour antigen and are able to control tumour growth.

#### 3.3.2 The dark side of TCR cross-reactivity

TCR cross-reactivity undoubtedly opens large avenues for developing more potent cancer therapies. However, there are important bottlenecks to consider. The possible side effects in immunotherapy, especially in adoptive T cell therapies, where optimised TCRs with high affinity against a certain peptide are administered to patients is a serious issue. Side effects with these engineered TCRs are not rare, due to the strong interaction between the TCR and its target. Very low expression levels of the antigen in healthy tissue, which was initially dismissed as potentially dangerous led to severe consequences (153, 154). This on-target toxicity is not that unexpected (**Figure 1D**). However, overlooking off-target effects due to TCR cross-reactivity can have similarly severe and fatal adverse effects as it was observed in the case of anti-MAGE-A3 TCR engineered T cells. Due to its restrictive expression to immune privileged sites such as placenta and testis which lack the expression of HLA molecules, MAGE-A3 was considered a genuinely tumour-specific target, since it is found to be overexpressed in multiple tumours. This *bona fide* target attracted the attention and promptly immunotherapies that target this molecule were developed. Contrary to the expectations, severe cases of toxicity were observed, despite the lack of antigen expression in any of the tissues affected. In the first of two well-known incidents, engineered anti-MAGE-A3_112–120_ (KVAELVHFL) T cells were adoptively transferred to cancer patients after nonmyeloablative lymphodepletion, who then received high doses of IL-2. This led to severe neurological damages, and even to a patient death. This fatal toxicity was attributed to a cross-reactivity of the effector TCRs with a MAGE-A12 sequence (KMAELVHFL) which has a superior binding affinity for HLA-A*0201 than MAGE-A3_112–120_. MAGE-A12 was found *a posteriori* to be expressed in the brain (153). In the second, even less predictable case, engineered lymphocytes with affinity-enhanced TCRs against the HLA-A*01-restricted MAGE-A3_168-176_ peptide (EVDPIGHLY) drove cardiotoxicity and patient death due to recognition of an unrelated peptide derived from the muscle protein titin (ESDPIVAQY) which is presented by cardiomyocytes (155, 156) (**Figure 1D**). Experimental and computational tools for prediction of potential toxicities have been improved since then and will be presented in section 4.

In general, therapeutic cancer vaccines are safe and no severe side effects have been observed to date. This may arise from the relatively low affinity of the induced T cells. The potential of MoAs to be used in immunisation approaches against tumour antigens, renders caution to what kind of side effects can arise. In a recent study, Gil-Cruz et al. showed that microbiota-derived peptide mimicry can induce lethal cardiomyopathy through the activation of heart-specific (MYH6-specific TCR) Th17 CD4^+^ T cells (157). In their mouse model, cross-reactive CD4^+^ T cells are primed in the intestine and later circulate and infiltrate the myocardium where they can damage myosin-expressing cells. In the context of checkpoint inhibition, it is tempting to speculate that not only self-, but also cross-reactive pathogen-specific T cells could be responsible for driving lethal cases of myocarditis that were observed in some patients (158, 159). The large number of auto-immune diseases that are associated with pathogen infection itself (section 2) demonstrate the delicate balance in the selection of these MoAs for therapeutic intervention.

## 4 Assessing TCR cross-reactivity: experimental evidence, *in silico* predictions and the need for high through-put testing platforms

A number of the examples of TCR cross-reactivity discussed so far have been brought to light using *in vitro* systems based on the testing of T cell activity against synthetic peptides. In early works, epitopic peptides of interest were modified by introducing aa substitutions at various positions. Later advances, supported by increased automatization of peptide synthesis, led to the development of synthetic peptide libraries. One common approach is to generate combinatorial (sub)libraries of peptides with each of the 20 aa fixed at one position while all other positions can be occupied by all other aa (160). Such approach can theoretically generate all possible aa sequences for a given peptide length and allows screening of up to 10^12^ peptides. *In vitro* testing of agonists or antagonists´ effects on T cell activity can be performed either by measuring cytokine secretion, killing of loaded target cells (for CD8^+^ T cells), or proliferation (for CD4^+^ and CD8^+^ T cells) (32, 161). Once a library has been shown to activate the T cell of interest, sub-libraries can be consecutively tested until the sequence(s) responsible for cross-reactivity is (are) identified. Subsequent database search can finally reveal whether the random peptide is indeed part of a known protein.

Together with the development of TCR engineering and adoptive transfer therapies, currently most advanced in the oncology clinical setting, high through-put and comprehensive approaches for testing TCR cross-reactivity have become mandatory for pre-clinical development. The main interest here is to assess TCR-mediated toxicity, i.e. the potential of transferred T cells to exert deleterious effects *in vivo* via recognition of non-related pMHC expressed on healthy tissues (**Figure 1D**). This is particularly relevant when the TCR has been manipulated for increasing its affinity to the cognate pMHC or has been obtained from HLA-unmatched donors (allorestricted). Challenges for the safe use of engineered TCRs in solid tumours have been very recently reviewed (162), and we have presented examples of fatal toxicities in section 3.3. In the context of clinical development, *in vitro* testing of T cell reactivity against random peptide sequences, as mentioned above, is the most straight-forward approach to assess cross-reactivity. DNA-tagged pMHC multimers, which allow to address TCR-pMHC affinity more easily is an elegant alternative method (163, 164). From the point of view of experimental feasibility, all these assays require high amount of material (e.g. T cell clones), which might be circumvented by modern methods. TCR cloning and subsequent transfer in reporter cells or MHC-matched PBMCs, and possibly the use of soluble TCRs and yeast pMHC libraries can overcome the aforementioned limitations (21). By titrating the peptide concentrations, TCR affinities can be more precisely assessed.

Which threshold of reactivity will lead to *in vivo* toxicity is likely impossible to predict with high accuracy and might even vary between individuals. One weakness of synthetic peptide testing is that recognition of a particular sequence by a certain TCR as measured *in vitro* cannot ultimately predict *in vivo* reactivity, since it is unknown whether this aa sequence is indeed processed and to which extent it is presented on body tissues. More sophisticated platforms try to overcome these limitations. First, testing primary normal cells from a range of organs representing essential human tissues (e.g. cardiovascular, gastrointestinal, brain, liver, among other systems) and/or a panel of tumour cell lines will assess potential off-target recognition (22, 165). Second, alloreactivity against MHC-mismatched cell lines can also be assessed (22, 166). As an example, reactivity of a TCR specific for a MAGEA4-derived epitope presented by HLA-A*0201 was found to recognise HLA-A*0205 (in the absence of MAGEA4), indicating alloreactivity; hence, patients bearing the HLA-A*0205 allelic product should be excluded from the clinical study using this TCR (22). Third, recognition of similar, but not identical synthetic peptides (containing aa substitutions), can also be tested *in vitro*, and the occurrence of potentially recognised sequences in the human proteome predicted. This combined approach could advantageously replace combinatorial peptide libraries (167).

Lastly, a comprehensive view of all peptides presented by MHC molecules in normal cells is needed and of utmost importance. The typical experimental setting for assessing the MHC ligand “landscape”, is to perform peptide immunoprecipitation followed by mass spectrometry analysis. First milestones steps have been engaged, with the Human Immunopeptidome Project (HIPP) and the HLA ligand atlas which both aim at deciphering the entire MHC-ligandome landscape of human healthy tissues (168, 169). In addition, quantitative analysis of peptide presentation by mass spectrometry has become possible. Using this method, it was recently shown that a peptide derived from collagen type VI A3 is present in 41% of the tumour samples analysed at an average of 228 (max 1928) copies per cell, but only in 6% of the normal tissues with an average of 28 copies (max of 49) per cell (165).

All these approaches are so far imperfect, since it cannot be excluded that an organ subpart, or specialised cells at a certain stage of differentiation or activation, may be targeted by cross-reactive T cells. As discussed earlier, the recognition by MAGE-A3 specific T cells of a titin-derived peptide expressed only in beating cardiomyocytes showed to be fatal for treated patients (155, 156). However, combining and refining them will decrease the chance of unexpected *in vivo* TCR cross-reactivity and toxicity. Possibly, tissue engineering and the development of 3D *in vitro* culture systems which better recapitulate the complexity of human organs and can be used in T cell assays might become a relevant addition to the testing pipelines.

In complement to experimental approaches, many efforts are ongoing for developing reliable *in silico* pipelines for predicting T cell cross-reactivity. It should be noted that many of such tools are not developed specifically for addressing cross-reactivity, but more generally to predict peptide immunogenicity (170). In principle, two aspects can be investigated: on the one hand, the probability for a peptidic sequence to be presented by various MHC allelic products, and on the other hand, the interaction of a specific TCR with a pMHC complex.

Regarding peptide MHC binding, the simplest strategy would be to start from the original peptide and deduce which altered sequence could or not bind to the presenting MHC allelic product. NetMHC and syfpeithi, which are essential publicly available tools, can deliver robust MHC-binding predictions, but they cannot directly interrogate TCR cross-reactivity. In addition, immunogenicity prediction tools based on aa properties (size, charge, aromaticity, gravy score) are also being developed (171, 172). In the tool available at the Immune Epitope Database (IEDB) (173), TCR preferences were deduced from the study of 600 immunogenic and 181 non-immunogenic 9mer peptides: the authors found out that peptides containing aromatic and large side chains aa (in particular phenylalanine) were preferentially recognised by T cells, and that positions 4-6 were the most critical, confirming previous findings. The task is much more complex when addressing the direct binding of a specific TCR to pMHC (see also section 1). Current approaches aiming at modelling such interactions in 3D are based on x-ray crystallography data (174–176). In addition, pMTnet, NetTCR and ERGO are neural networks that predict pMHC-TCR (CDR3 regions of the TCR β, and more recently, α chains) and are in continuous refinement (177–179).

*In silico* tools rely on the exploitation of experimental data. Hence, *in vitro* testing of e.g. peptide library scanning is not only useful for current assessment of T cell cross-reactivity, it is also needed for training and improving prediction tools. In this respect, repository of TCR-pMHC interactions and affinities, as well as 3D information, such as those available at the IEDB, Altered TCR Ligand Affinities and Structures (Atlas) (180), Structural T-cell Receptor Database (STCRDab) (181) or TCR associated with pathology conditions (McPAS-TCR) (182) and PMID databases are essential. Implementation of more information, in particular for rare MHC allelic products, is still necessary and will help improving the robustness of these approaches in the next years.

## 5 Concluding remarks

Cross-reactivity is a very smart property of our adaptive immune system to cope with the large pathogen universe. It also plays a significant role in pathological conditions as different as autoimmunity and cancer. While it has been longer discussed that virus- or bacteria-specific T cells are associated with some autoimmune diseases, more recent research is uncovering their role in cancer. This knowledge can be exploited for therapy in both diseases. Application of mimotopes in the treatment of autoimmune diseases will depend to a large extent upon their ability to suppress immunoreactivity, for instance by stimulating regulatory anti-inflammatory CD4^+^ T cells, or by directly inhibiting pathogenic cytotoxic CD8^+^ T cells. This is obviously a complex task, and identification and prediction of self-epitopes and mimotopes recognised by particular TCRs is, therefore, important to make such antigen-specific approaches successful in autoimmune diseases. In cancer immunotherapy, TCR cross-reactivity is becoming an essential consideration, not only for designing more efficient T cell-based treatments, but also for preventing severe side effects. Considering the ongoing personalisation of therapeutic approaches, the upstream TCR selection process needs to be speed up. The development of novel and refined prediction methods is of utmost importance, but is a challenging process due to the numerous aspects that can impact cross-reactivity.

## Author contributions

CG, AM, and RK conceived the review and jointly wrote the manuscript. AM prepared the figure which was reviewed by all authors. All authors contributed to the article and approved the submitted version.
